# Increased planting density combined with reduced nitrogen rate to achieve high yield in maize

**DOI:** 10.1038/s41598-020-79633-z

**Published:** 2021-01-11

**Authors:** Xiangbei Du, Zhi Wang, Weixia Lei, Lingcong Kong

**Affiliations:** Crop Research Institute, Anhui Academy of Agricultural Sciences, Hefei, 230031 Anhui Province People’s Republic of China

**Keywords:** Plant development, Environmental sciences

## Abstract

The combination effects of nitrogen (N) fertilizer and planting density on maize yield, N use efficiency and the characteristics of canopy radiation capture and radiation use efficiency are not well documented in the Huanghuaihai Plain region in China. A 2-year field experiment was conducted from 2017 to 2018 in a split plot design with two N levels (240 and 204 kg N ha^−1^) applied to main plots and three plant densities (67,500, 77,625 and 87,750 plants ha^−1^) allocated to sub plots. Our results show that a 30% greater plant density combined with a 15% lower N rate (basal N) enhanced N partial factor productivity (NPFP) by 24.7% and maize grain yield by 6.6% compared with those of the conventional high N rate combined with a low density planting management practice. The yield increase was mainly attributed to significantly increased kernel numbers and biomass. The increased intercepted photosynthetically active radiation (IPAR) was the primary factor responsible for the high productivity of maize at increased planting density under reduced N conditions. The results indicate that increase planting density with reduced basal N application might benefit maize cropping for achieving high yields and sustainable development of agriculture.

## Introduction

Maize (*Zea mays* L.) is a major staple crop species and accounts for 60% of global human consumption, livestock feed and raw materials for industrial purposes^1,2^. Nitrogen (N) is a key element for maize yield^3,4^. Previous studies have shown that increasing N application rate is important way to obtain high grain yield^5,6^. However, excessive fertilization is a severe problem that increases grain yield but reduces N use efficiency (NUE)^7–9^, increases costs, and causes severe environmental pollution^10,11^. Improving fertilizer utilization, especially with higher N use efficiency, is one of the essential strategies to achieve the goals of sustainable agriculture^12^. In addition to nitrogen, planting density is another key factor in determining maize yield^13–16^. Previous studies have shown that increasing density is among the major factors associated with increases in maize grain yield^13,17–21^ and N use efficiency^22,23^. However, increased density also increases the plants competition for light and nutrients^24^.

Farmers in China frequently use more N fertilizer than necessary for high maize yields. However, this approach does not increase the grain yield and in fact actually reduce economic benefits^25^. Therefore, it is imperative to optimize fertilizer management during the maize growth period, which can not only reduce N input but also improve grain yield and environmental sustainability^26,27^. While increased density generally stimulates crop productivity^28,29^, reduced N often has the opposite effect on yield^3,22^. Previous studies have shown that densification may compensate for the negative effects of reduced N on crop productivity^30^. Therefore, a reasonable increase of planting density and supplying adequate N application are important agronomic practices to increase maize grain yield.

Theoretically, maize yield depends on the total biomass accumulation and its partitioning to the grain. It is well known that biomass production is the product of intercepted photosynthetically active radiation (IPAR), which is a function of both canopy architecture and the leaf area index (LAI), that is converted into biomass (radiation use efficiency, (RUE))^31^. Previous studies have shown that plant density and N rates significantly affect canopy structure, leading to differences in the IPAR and RUE, which ultimately influence yield^28,32^. Greater yields associated with maize under different N rate and planting density can result from greater IPAR, RUE, or a combination of the two^33^. Previous studies has suggested that planting density and N application rate interact to obtain high grain yield and NUE^5,6,17,25^. However, to our knowledge, the combined effects of planting density with N application rate on maize canopy development and radiation capture and use efficiency use are not well documented.

Our objectives are to develop an optimum combination of an appropriate N rate and planting density aimed at achieving high yields with reduced inputs for environmentally friendly maize cropping. Thus, a 2-year field experiment was conducted to determine the combined impacts of planting density and N rate on maize yield, NUE and the characteristics of canopy radiation capture and radiation use efficiency. We expect that the results will provide new insight for understanding the mechanisms underlying the establishment of high-yield and high-efficiency maize cultivation and provide guidance for future maize cropping practices.

## Results

### Grain yield and yield components

With the exceptions of thousand-kernel weight and the harvest index (HI), ear number, number of seeds per ear, kernel number, yield and biomass were significantly affected by planting density and its interactions with N rate in 2 years (Table 1). Higher productivity was observed in the high density treatments. The number of seeds per ear in the D2 and D3 treatments was lower than that in the D1 treatment, but the ear number significantly increased, resulting in an increased number of kernels per unit area. The yield and biomass of N1D3 treatments were the highest in the six treatments. The N1D2, N1D3, N2D1, N2D2 and N2D3 treatments increased biomass by 4.8%, 7.3%, − 4.3%, 1.0% and 6.0%, respectively, and the grain yield increased by 4.7%, 8.1%, − 6.0%, 0.7% and 6.6%, respectively, compared to that in the CK, over two growing seasons.

**Table 1:** Yield and yield components of maize in different planting treatments in 2017 and 2018.

Year	Planting treatments	Ear number (ha^−1^)	Number of seed per ear	kernel number (m^2^)	Thousand-kernel weight (g)	Yield (t ha^−1^)	Biomass (t ha^−1^)	HI
2017	N1D1(CK)	59,448.3b	456.3a	2712.6bc	319.4a	8.6ab	14.2bc	0.52a
	N1D2	64,243.5ab	438.2ab	2815.2ab	317.1a	8.9a	15.0ab	0.51a
	N1D3	70,141.7a	420.4b	2948.8a	308.5b	9.2a	15.6a	0.51a
	N2D1	58,523.3b	445.2a	2605.5c	315.8ab	8.2b	13.4c	0.53a
	N2D2	63,546.2ab	432.5ab	2748.4ab	311.8ab	8.7ab	14.2bc	0.53a
	N2D3	69,937.5a	421.9b	2950.7a	306.2b	9.1a	15.2a	0.52a
	Analysis of variance							
	N rate (N)	ns	ns	ns	ns	*	ns	ns
	Density (D)	**	*	**	ns	*	**	ns
	N*D	*	*	*	*	*	*	ns
2018	N1D1(CK)	63,897.5b	454.9a	2906.7bc	320.8a	9.2ab	15.9b	0.50a
	N1D2	69,956.1ab	442.1ab	3092.8ab	315.6ab	9.7ab	16.5ab	0.51a
	N1D3	74,921.7a	432.6b	3241.1a	310.1b	10.2a	16.9a	0.52a
	N2D1	62,245.2b	446.6a	2779.9c	318.1ab	8.8b	14.9c	0.51a
	N2D2	68,794.9ab	437.8ab	3011.8ab	313.8ab	9.4ab	16.1ab	0.50a
	N2D3	73,969.2a	430.5b	3184.4a	309.9b	10.0a	16.9a	0.50a
	Analysis of variance							
	N rate (N)	ns	ns	ns	ns	ns	ns	ns
	Density (D)	**	*	**	ns	*	**	ns
	N*D	*	*	*	*	*	*	ns

Values followed by different letters within a column are significantly different (*P* < 0.05).

*HI* harvest index, *ns* no significant effects.

*Significant effects at *P* < 0.05.

**Significant effects at *P* < 0.01.

### Crop N uptake and N use efficiency

**Figure 1:**
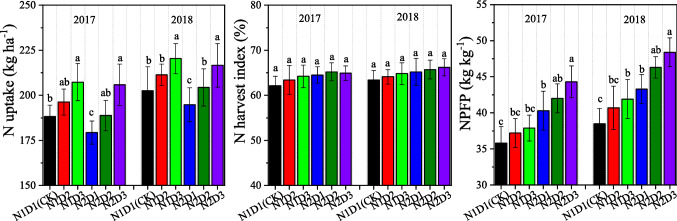
N uptake, N harvest index and N partial factor productivity of maize (NPFP) under different planting treatments in 2017 and 2018. Error bars indicate standard errors of replicates. Means followed by the same letter are not significantly different among different planting treatments at *P* < 0.05.

N uptake was significantly affected by density and its interactions with N rate both in 2017 and 2018, and NPFP was significantly affected by N rate and its interactions with density in 2 years (Table 2). Across both years, the N1D2, N1D3, N2D1, N2D2 and N2D3 treatments increased the N uptake by 4.3%, 9.4%, − 4.2%, 0.6% and 8.1%, respectively, and increased the NPFP by 4.8%, 7.3%, 12.6%, 18.9% and 24.7%, respectively, compared to that of the CK (Fig. 1). There were no significant interactions in N harvest index (NHI) between planting treatments.

**Table 2:** Variance analysis for the N rate and density and their interactions effect on N uptake and use efficiency, and radiation capture and use efficiency.

Analysis of variance	N uptake	NPFP	NHI	LAI_max_	*f*PAR	IPAR	*k*	RUE
**2017**								
N rate (N)	ns	*	ns	ns	ns	ns	ns	ns
Density (D)	*	ns	ns	*	*	*	ns	ns
N*D	*	*	ns	*	*	*	*	*
**2018**								
N rate (N)	*	*	ns	ns	ns	ns	ns	ns
Density (D)	*	ns	ns	*	*	*	ns	ns
N*D	*	*	ns	*	*	*	*	*

*NPFP* N partial factor productivity, *NHI* N harvest index, *LAI*_*max*_ maximum leaf area index, *fPAR* fraction of PAR intercepted, *IPAR* cumulative intercepted PAR, *k* light extinction coefficient, *RUE* radiation use efficiency, *ns* no significant effects.

*Significant effects at *P* < 0.05.

**Significant effects at *P* < 0.01.

### Canopy structural characteristics

**Figure 2:**
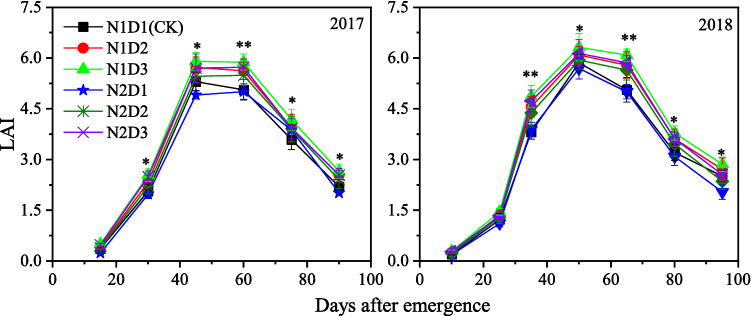
Maize leaf area index (LAI) trends during growing season as affected by planting treatments. Each data point is the mean ± S.E. of three replications with * and ** are significant at *P* < 0.05 and *P* < 0.01, respectively.

As shown in Fig. 2, maize LAI increased dramatically during the initial growth stage, peaked at the same time and then declined with leaf senescence in all treatments. The LAI of maize was distinctly affected by density and its interactions with N rate both in 2017 and 2018, and a relatively high LAI was observed in the high density treatments (Table 2). Averaged over two years, the peak values of the LAI for the N1D2, N1D3, N2D1, N2D2 and N2D3 treatments were 5.7%, 9.5%, − 4.1%, 2.4% and 6.4% higher than the value of the CK, respectively.

**Figure 3:**
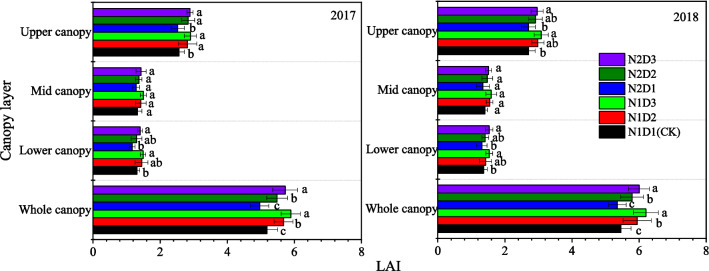
Vertical distribution of LAI in the canopy of maize in different planting treatments. The data were averaged measurements on 45 and 60 days after emergence (DAE), 2017 and 50 and 65 DAE, 2018. Upper canopy, middle canopy and lower canopy of maize mean top-two leaves above the ear leaf, the ear leaf and two leaves above and below it, and two leaves below the ear leaf-bottom leaf, respectively. Each data point is the mean ± S.E. of three replications. Values followed by different letters within a column are significantly different (*P* < 0.05).

Planting treatments also altered the vertical distribution of the maize LAI at the tasseling and silking stages (Fig. 3). There was little difference in the LAI of the middle and lower canopies among the six treatments. By contrast, marked differences were noted in the LAI of the upper and whole canopies among the six planting treatments. The LAI of the high density treatments increased for the upper and whole canopy but showed no significant difference for the middle and lower canopy compared to that in the CK.

### PAR interception

**Figure 4:**
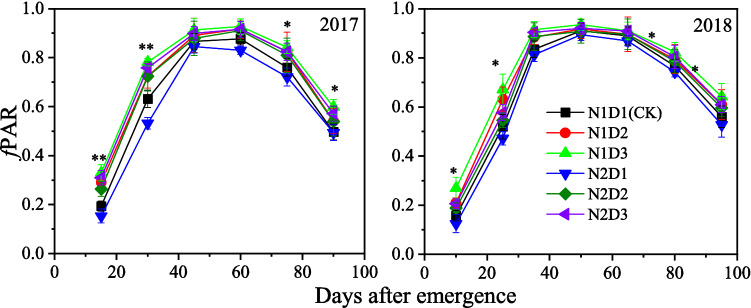
Changes in fraction of PAR intercepted (*f*PAR) by maize affected by planting treatments during the growing season. Each data point is the mean ± S.E. of three replications with * and ** are significant at *P* < 0.05 and *P* < 0.01, respectively.

The *f*PAR of maize was distinctly affected by density and the interactions between density and N rate both in 2017 and 2018 (Table 2). The *f*PAR in the high density treatments had higher *f*PAR than that in the CK, throughout the growing season (Fig. 4). Compared to that of the CK, the mean *f*PAR of the N1D2, N1D3, N2D1, N2D2 and N2D3 treatments increased by 8.1%, 13.1%, − 5.3%, 5.7% and 9.0%, respectively, averaged over 2 years.

**Figure 5:**
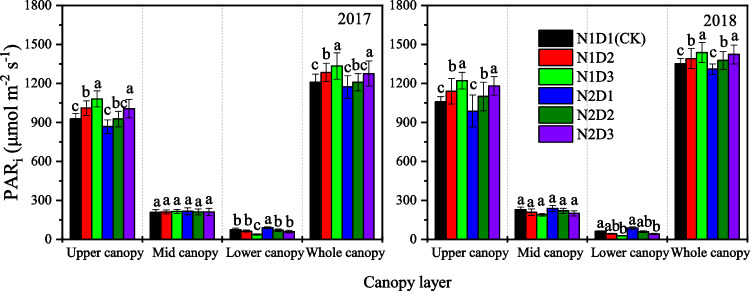
Vertical distribution of incident PAR intercepted (PAR_i_) under different planting treatments at tasseling and silking stage. The data were averaged measurements on 45 and 60 DAE, 2017 and 50 and 65 DAE, 2018. Upper canopy, middle canopy and lower canopy of maize mean top-two leaves above the ear leaf, the ear leaf and two leaves above and below it, and two leaves below the ear leaf-bottom leaf, respectively. Each data point is the mean ± S.E. of three replications. Values followed by different letters within a column are significantly different (*P* < 0.05).

Planting treatments also had significant effects on the amount of PAR intercepted (PAR_i_) by the maize canopy (Fig. 5). The PAR_i_ in the upper canopy decreased in the order of N1D3 > N1D2 > N2D3 > N2D2 > CK > N2D1 for both years. In contrast, the PAR_i_ in the lower canopy exhibited the opposite trend, with the order of N2D1 > CK > N2D2 > N2D3 > N1D2 > N1D3 for both years. Little difference in PAR_i_ was detected in the middle canopy. The differences in the PAR captured by the maize canopy in the different planting treatments could be attributed to the PAR captured by the upper canopy. Compared to that for the CK, the whole-canopy PAR_i_ for the N1D2, N1D3, N2D1, N2D2 and N2D3 treatments were 4.6%, 8.3%, − 3.0%, 1.0% and 5.4% greater, respectively, averaged across both years.

### Canopy light extinction

**Figure 6:**
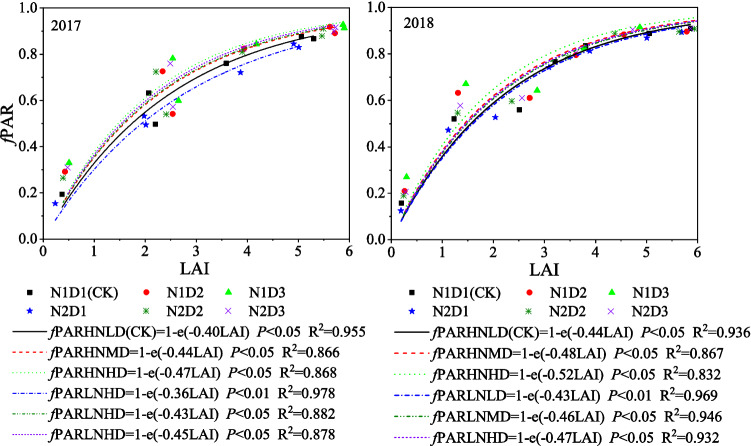
Relationship between the fractions of PAR intercepted (*f*PAR) and the leaf area index (LAI) of maize in different planting treatments.

There was a close exponential relationship between the *f*PAR and LAI of maize in the different planting treatments (Fig. 6). The extinction coefficient (*k*) was notably affected by the interactions between density and N rate both in 2017 and 2018 (Table 2). The *k* of the high density treatments were consistently higher than that of the CK across the two years. The equations depicted in Fig. 6 show that the *k* values of the CK, N1D2, N1D3, N2D1, N2D2 and N2D3 treatments were 0.42, 0.46, 0.50, 0.40, 0.45 and 0.46, respectively, averaged over 2 years.

### Radiation capture and radiation use efficiency

The IPAR and RUE of maize were significantly affected by the interactions between density and N rate both in 2017 and 2018 (Table 2). The D2 and D3 treatments intercepted significantly more PAR than did the D1 treatments. Compared to that of the CK, the mean IPAR of the N1D2, N1D3, N2D1, N2D2 and N2D3 treatments increased by 6.2%, 14.3%, − 4.9%, 4.2% and 12.4%, respectively, averaged over 2 years (Table 3). The increased IPAR could be attributed to the significantly higher *f*PAR throughout the maize growth period. The RUE of maize under the D3 treatments was consistently lower than that under the CK. When averaged over both years, the RUE values of the N1D2, N1D3, N2D1, N2D2 and N2D3 treatments were 1.4%, 5.3%, 1.2%, 3.3% and 5.1% lower than the value of the CK, respectively.

**Table 3:** Cumulative intercepted photosynthetically active radiation (IPAR), and radiation use efficiency (RUE) of maize in four planting treatments in 2017 and 2018.

Year	Planting treatments	IPAR (MJ m^−2^)	△IPAR (%)	RUE (g MJ^−1^)	△RUE (%)
2017	N1D1 (CK)	430.7bc		3.30a	
	N1D2	456.8b	6.06	3.28a	− 0.53
	N1D3	493.6a	14.60	3.17ab	− 3.97
	N2D1	412.6c	− 4.20	3.25a	− 1.52
	N2D2	445.1b	3.34	3.19ab	− 3.16
	N2D3	483.2a	12.19	3.15b	− 4.40
2018	N1D1 (CK)	465.5bc		3.42a	
	N1D2	495.2c	6.38	3.34ab	− 2.23
	N1D3	530.4a	13.94	3.19b	− 6.68
	N2D1	439.9c	− 5.50	3.39ab	− 0.81
	N2D2	488.5b	4.94	3.30ab	− 3.49
	N2D3	524.3a	12.63	3.22ab	− 5.87

Values followed by different letters within a column are significantly different (*P* < 0.05).

*ΔIPAR and ΔRUE* change of IPAR and RUE in the planting treatments in relation to the CK.

### Relationships between yield, N uptake and N use efficiency and radiation capture and radiation use efficiency

The relationships between the maize grain yield and yield components, N uptake and N use efficiency, and radiation capture and radiation use efficiency were investigated (Table 4). The data showed that the grain yield and biomass were significantly and positively correlated with the N uptake, LAI, *f*PAR and IPAR but were no correlated with the RUE. These correlations suggested that maize productivity was limited by the relatively low LAI leading to little *f*PAR and relatively low radiation capture.

**Table 4:** Correlation coefficients among maize yield, kernel number, thousand kernels weight, biomass, N uptake, N partial factor productivity (NPFP), N harvest index (NHI), leaf area index (LAI), mean fraction of PAR intercepted (*f*PAR), cumulative intercepted PAR (IPAR) and radiation use efficiency (RUE) for different planting treatments in 2017 and 2018.

Index	Yield	Kernel number	Thousand kernels weight	Biomass	N uptake	NPFP	NHI	LAI	*f*PAR	IPAR	RUE	k
Yield	1.000											
Kernel number	0.978**	1.000										
Thousand kernels weight	– 0.318	– 0.507	1.000									
Biomass	0.987**	0.963**	– 0.307	1.000								
N uptake	0.961**	0.981**	– 0.491	0.968**	1.000							
NPFP	0.44	0.506	– 0.492	0.427	0.417	1.000						
NHI	0.349	0.442	– 0.576	0.345	0.354	0.927**	1.000					
LAI	0.696*	0.793**	– 0.740**	0.684*	0.798**	0.202	0.26	1.000				
FPAR	0.708**	0.790**	– 0.679*	0.722**	0.821**	0.197	0.244	0.964**	1.000			
IPAR	0.941**	0.985**	– 0.593*	0.940**	0.980**	0.472	0.432	0.857**	0.853**	1.000		
RUE	– 0.028	– 0.221	0.890**	0.014	– 0.189	– 0.202	– 0.314	– 0.626*	– 0.513	– 0.328	1.000	
k	0.908**	0.929**	– 0.48	0.908**	0.943**	0.316	0.322	0.840**	0.895**	0.931**	– 0.218	1.000

n = 12, R_0.05_ = 0.576, R_0.01_ = 0.707.

*Significance of correlation at 0.05 level.

**Significance of correlation at 0.01 level.

## Discussion

Our results showed that reducing the N rate by 15% from 240 to 204 kg ha^−1^ resulted in significantly increased grain yield (by 5.1–6.8%) when combined with a 30% higher planting density (Table 1), which is consistent with the result of a previous study; the maize yield loss from reduced N input could be compensated by increased density^5,14,34^. We also found that increased planting density had positive effects on maize yield, with a relative yield increase of 3.8–8.8% recorded on the conventional high N rate.

Maize yield was determined by kernel number per unit area and thousand-kernel weight; the kernel number per unit area was considered the primary determinant^35^. In the present study, the increased yields in N2D3 treatments were mainly attributed to the increased kernel number. The results revealed that the N rate and density interaction significantly affected the kernel number and was strongly associated with yield (Table 4), which was consistent with the result of previous works^13,19,23^. For the high density treatments, a higher ear number could compensate for the negative effects of a lower kernel number per ear. As such, the kernel number increased by 9.6–10.6%, which resulted in increased grain yields. Jiang et al.^36^ reported that for smaller groups, maize yield increased mainly because of increased ear numbers. It is worth noting that if only the N rate was reduced without increasing the planting density, the maize population might not be not large enough to achieve high yields (Table 1).

**Figure 7:**
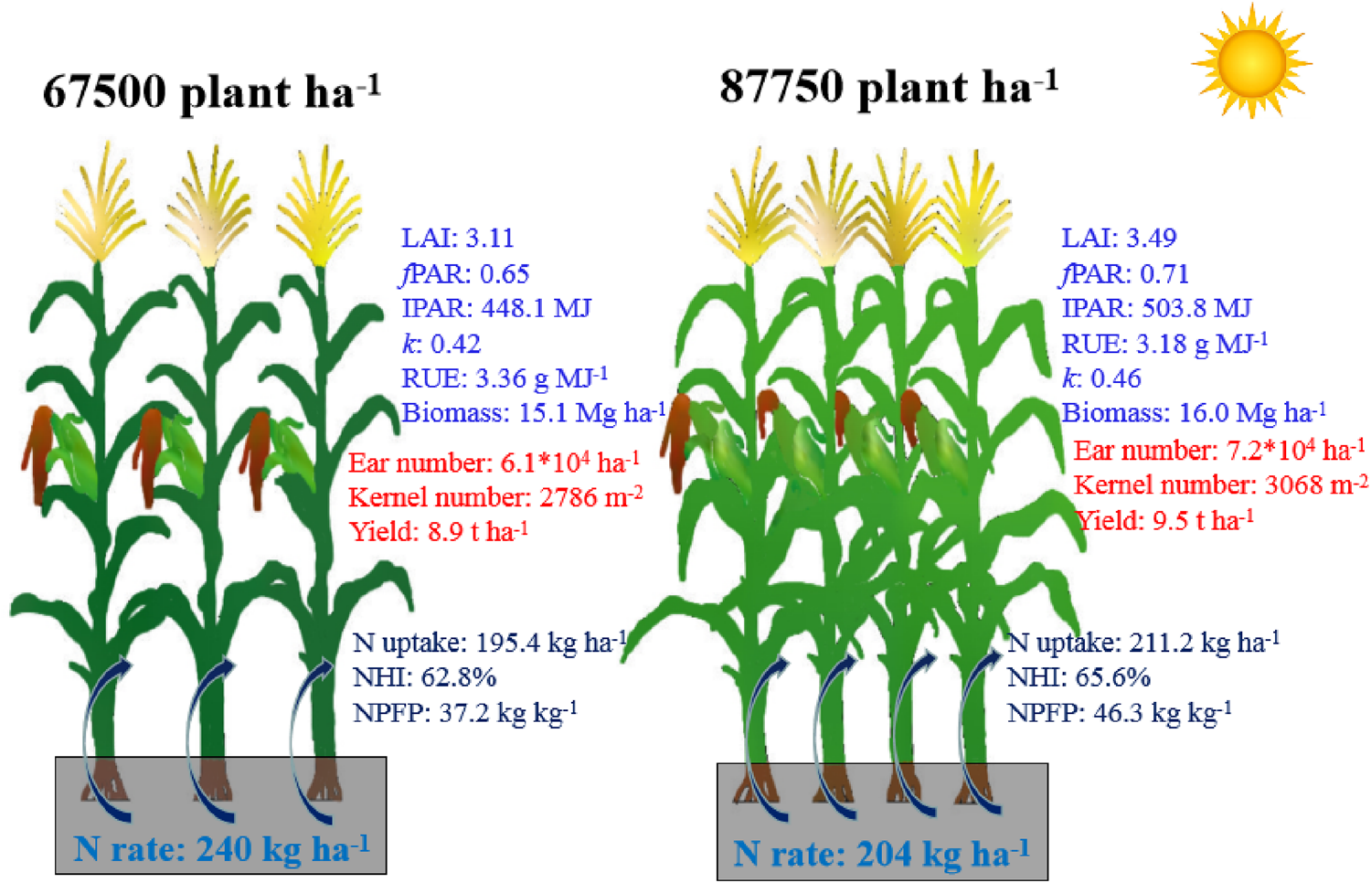
Schematic representation of the processes involved in the canopy development, radiation capture and radiation use efficiency, N uptake and N use efficiency and yield. All data in the figure were averaged by two experiment years. *LAI* leaf area index, *fPAR* fraction of PAR intercepted, *IPAR* cumulative intercepted PAR, *RUE* radiation use efficiency, *k* light extinction coefficient, *NHI* N harvest index, *NPFP* N partial factor productivity.

It is well known that crop yield formation depends on biomass accumulation and allocation to the grain. Numerous studies have demonstrated that yield increases could be achieved from enhanced biomass production, harvest index improvements or both^23,30,37^. In our study, there was an increase of 6.0–7.3% in the biomass in the N2D3 treatments compared to the CK, while the HI was generally unchanged (Table 1). The high yields in the N2D3 treatment were mainly derived from increased biomass accumulation (Fig. 7).

Biomass is the product of radiation intercepted by the canopy multiplied by the RUE. An increase in yield often results from increased radiation capture, more efficient use, or a combination of two^38^. The increased biomass production was almost exclusively caused by the increase in the IPAR (12.2–12.6%) rather than changes in the RUE (decrease of 4.4–5.9%) (Table 3). The results of our study demonstrated that the yield advantage of maize was mainly attributed to the increased IPAR. Our results corresponded with the typical explanations for increased grain yields at optimum density resulting from increased radiation interception^39,40^. This increased radiation capture during the critical period has been thought to be the primary reason for yield increases in many crops^41–43^. The lower IPAR was the main reason that the whole-canopy photosynthetic capacity was weaker in conventional traditions maize planting practices used by farmer (Fig. 7).

The effects on radiation capture were explained by the improved maize canopy, i.e., quicker early canopy establishment and greater interception during the whole growth period. Understanding the canopy architecture of maize is important for manipulating plant density for continuous maize improvement^16,44,45^. Compared to that under the CK, the maize plant growth under the N2D3 treatments presented 11.8–12.3% greater LAI values. Early and rapid canopy establishment allows maize to take advantage of higher IPAR pre-canopy closure^46,47^, where the greatest amount of radiation is available in June and July (Table 5). The greater LAI resulted in a greater *f*PAR, compared with that of the CK, and an 6.3–11.9% increase in the *f*PAR was observed for the entire growth period in the N2D3 treatments (Fig. 4), as well as an increased in cumulative intercepted solar radiation for a similar growth period.

**Table 5:** Monthly rainfall, monthly photosynthetically active radiation (PAR) and daily average temperature at the experimental field in 2017 and 2018.

Meteorological data	Year	Jun	Jul	Aug	Sep	Total
Rainfall (mm)	2017	80.1	130.0	260.7	283.5	754.3
	2018	210.1	189.3	271.4	31.9	702.7
Average temperature (℃)	2017	25.3	29.7	27.1	22.4	26.1
	2018	26.2	28.7	28.4	23.2	26.6
PAR (MJ m^−2^)	2017	259.9	297.7	234.0	180.3	971.9
	2018	277.4	270.6	244.2	191.5	983.8

PAR was estimated by multiplying solar radiation by 0.5.

The RUE value determined in the present study ranged from 3.15 to 3.42 g MJ^−1^, which was similar to values in the literature for maize in different parts of the world (3.0–4.0 g MJ^−1^)^48–51^. Our results showed that dense planting combined with a 15% reduced N rate resulted in lower RUE that was lower than that of the CK. The main reason for the effects of planting treatment on maize RUE was the vertical distribution of the PAR_i_ in the lower canopy layer. In the present study, both the absolute value of the LAI (Fig. 3) and the amount of PAR_i_ captured (Fig. 5) in the upper canopy layer under the N2D3 treatment was greater than those under the CK, which led to poor light distribution through the canopy (higher *k*), and relatively more light captured by the upper canopy has been suggested to reduce the whole plant photosynthetic efficiency, which in turn decreases the RUE^52,53^. Because a larger fraction of shaded leaves may lead to a higher ineffective photosynthetic area and canopy respiratory consumption, the whole canopy conversion efficiency is reduced especially at a high LAI. As a result, the N2D3 treatment had a relatively lower RUE.

Excessive N applied at the basal time is the main constraint on the crop NUE under conventional planting practices in China^54,55^. Dense planting with a reduced N rate was believed to improve not only grain yield but also N use efficiency^7,30,56^. In our present study, reduced basal N input significantly increased NPFP compared to CK (Fig. 1). A reduction in basal N application could significantly reduce N loss during the lower demand early growth stage of maize seedlings^54,57^. The results from this study suggest that both higher yield and NUE may be achieved in maize production by optimizing N application and density interaction management practices.

From the perspective of our study, improvement of radiation interception through producing a reasonable canopy structure is an effective strategy for enhancing crop yields (Fig. 7). As such, increasing the planting density is a suitable agronomic practice for manipulating the structure of the population and canopy. Thus, increasing density is recommended to further increase grain yields. In our environment, a poor RUE was detected in the high density treatments. Furthermore, agronomic measures (e.g., row spacings, canopy types, use of growth regulators) should be optimized to synchronously increase the IPAR and the RUE^15,45,58–60^. Moreover, reducing the amount of N applied is recommended to improve N use efficiency, as excessive N input is very common in the country^54^. Our results indicate that increasing planting density combined with less basal N input can be a good technique for high-yield and environmentally friendly maize cropping in the Huanghuaihai Plain maize region in China.

## Conclusions

This study demonstrates that high maize grain yield and high NUE could be simultaneously achieved by 30% increase in planting density combined with a 15% reduction in the basal N applied. Increased grain yields were associated with increased sink capacity, and increased radiation capture was the primary factor responsible for the high productivity. In conclusion, it is possible and sustainable to maintain high grain yields with a reduction in basal N fertilization by increasing plant density in maize cropping systems in the Huanghuaihai Plain maize region in China.

## Materials and methods

### Experimental site

Field experiments were conducted in 2017 and 2018 at the experimental station of the Crop Research Institute, Anhui Academy of Agricultural Sciences (33° 11′ N, 116° 86′ E) in Huaiyuan County, Anhui Province, China. The soil type or the field is a Shajiang black soil. Soil samples from the 0 to 20 cm soil layer were taken before fertilizer application during each growing season at the beginning of the field experiments. The organic matter (via the K_2_Cr_2_O_7_–H_2_SO_4_ oxidation method), total N (via the Kjeldahl method), available phosphorus (via the Olsen method), and available potassium (according to the ammonium acetate extraction method) in the top 20 cm of the soil were 25.7 g kg^−1^, 1.6 g kg^−1^, 17.9 mg kg^−1^, and 159.3 mg kg^−1^ in 2017 and 27.3 g kg^−1^, 1.6 g kg^−1^, 18.6 mg kg^−1^, and 168.4 mg kg^−1^ in 2018, respectively. Daily records of the meteorological data, including the solar radiation, temperature and rainfall, were obtained from a weather station located adjacent to the experimental field. Daily incident PAR (400–700 nm) was calculated as 50% of the total daily solar incident radiation^61^. A summary of the monthly environmental meteorological data during the maize growing seasons in 2017 and 2018 was listed in Table 5.

### Experimental design and field management

The field experiment plots were arranged in a split plot design with three replicates. The main plots were assigned to two N fertilization treatments (N1: 240 kg ha^–1^ and N2: 204 kg ha^–1^), and subplots were assigned to three planting densities (D1: 67,500 plants ha^−1^, D2: 77,625 plants ha^−1^ and D3: 87,750 plants ha^−1^). The size of each plot was 10.0 × 7.2 m (12 rows spaced 60 cm apart). The conventional high N rate combined with a low density planting management practice (N1D1) for high yield as control (CK). The spacing between plants within a row depended on the plant density. Phosphate (superphosphate, 12% P_2_O_5_) and potassium (potassium sulfate, 60% K_2_O) fertilizers were applied as a basal fertilizer at 90 and 120 kg ha^–1^, respectively. For the N1 treatments, N (urea, 46.4% N) was applied as a basal fertilizer at 120 kg ha^–1^ before sowing and top-dressed at the V8 stages at a rate of 120 kg ha^–1^. For the N2 treatments, N (urea, 46.4% N) was applied as a basal fertilizer at a rate of 84 kg ha^–1^ before sowing and then top-dressed at the V8 stages at 120 kg ha^–1^. Detailed information about the N application rates and timing and planting density is listed in Table 6.

**Table 6:** N fertilization application and planting density for each planting treatments.

Planting treatments	N rate and timing (kg ha^−1^)			Planting density	
	Total	Basal	Top-dressed	Spacing (cm)	Plant number (plant ha^−1^)
N1D1 (CK)	240	120	120	60.0 × 24.6	67,500
N1D2	240	120	120	60.0 × 21.5	77,500
N1D3	240	120	120	60.0 × 19.0	87,750
N2D1	204	84	120	60.0 × 24.6	67,500
N2D2	204	84	120	60.0 × 21.5	77,500
N2D3	204	84	120	60.0 × 19.0	87,750

The cultivar Zhengdan 958 which is widely grown in the Huanghuaihai Plain maize region in China, was used^62^. The previous crop grown in the field was winter wheat. After the harvest of the winter wheat, the fields were prepared with a rotary tiller to till the soil to a depth of 15 cm for maize sowing. The maize seeds were hand-planted, with two seeds per hill, on 8 June 2017 and 11 June 2018, respectively. The seedlings were then thinned to one seedling per hill to maintain the desired plant densities at 10 days after emergence. Crop management was in accordance with local high-yield cultural practices. There was no significant difference in the crop development (e.g., time of anthesis and black layer maturity) between the planting treatments. Maize was harvested on 30 September and 29 September 2017 and 2018, respectively.

### Sampling and measurements

#### Plant biomass and N analysis

Five maize plant samples per plot were collected at 15-day intervals from 25 June to 30 September 2017 and from 23 June to 25 September 2018. The sampled maize plants were cut at the soil surface and separated into leaves, stems and ear shoot components. The dry weight of each component was determined after drying for 72 h at 80ºC in a forced-air drying oven. The plant samples were then milled, and the N concentration was analyzed based on the Kjeldahl method^63^. N uptake was calculated by multiplying the N concentration by the dry weight. The NHI was calculated as the N uptake in the grain divided by the total N accumulation in aboveground plants. N use efficiency was defined as the N partial factor productivity (NPFP), which was determined by the grain yield per unit of N applied (kg kg^−1^).

#### Leaf area index

The green LAI of the plant population for vertical canopy layers (e.g., soil surface, two leaves below the ear, two leaves above the ear, and 0.1 m above the maize canopy) was measured on the plant sample times. Lamina legth (L) and maximum lamina width (W) were recroded and leaf area (A) was calculated using the following equation:

1 $${\text{A}} = 0.75 \times L \times W,$$

LAI was calculate as the sum of the areas of green leaves per unit area of land by plants^64^.

#### Determination of photosynthetically active radiation

The PAR was measured at different vertical canopy layers (e.g., at the soil surface, two leaves below the ear, two leaves above the ear, and 0.1 m above the maize canopy) in each plot using a SunScan Canopy Analysis System at noon on clear days. At least five readings of photosynthetic photon flux density were taken at each layer. The line quantum sensor was held in different directions but always parallel to the earth’s surface. The data were recorded in 15-day intervals from 25 June to 19 September, 2017 and from 23 June to 20 September, 2018.

#### Radiation capture and radiation use efficiency

The fraction of photosynthetically active radiation (*f*PAR) of each treatment was then calculated using the following formula:

2 $$fPAR = 1 - \frac{{PAR_{b} }}{{PAR_{a} }},$$

where PAR_a_ and PAR_b_ are the above- and below-canopy PAR, respectively. The values of the *f*PAR for each day were estimated by fitting polynomial functions between the measured *f*PAR and days after emergence^43,65^.

The IPAR intercepted (*∑*IPAR) between two measurements was calculated using the following equation^66^:

3 $$\sum {{\text{IPAR}}} = \Omega \times 0.5 \times fPAR_{d} ,$$

where Ω is the summation of the total daily incident solar radiation between the investigation periods and *f*PAR_d_ is the daily fraction of PAR interception during the investigation periods.

The RUE (g MJ^−1^) was calculated by dividing the total biomass (g m^−2^) by the cumulative IPAR (MJ m^−2^) during the growth period as follows^43^:

4 $${\text{RUE}} = \frac{{{\text{Biomass}}}}{{{\text{IPAR}}}}$$

#### Yield and yield components

At maturity, plants within a quadrate 2.0 m long and 1.2 m wide (in the middle two rows) in each plot were harvested by hand to determine the grain yield in both seasons. Ears per square and kernels per ear were counted, and kernel numbers per square were determined. The thousand-kernel weight and grain moisture content were determined, and the yield was adjusted for 14.0% grain moisture content. The HI was calculated based on the grain yield and the total aboveground plant biomass at maturity.

### Statistical analysis

The data were statistically analyzed using SPSS 20.0 statistical software. The data are presented as the means ± standard errors. Figures were then constructed using Origin 2018 software.
